# Safety Evaluation of Silk Protein Film (A Novel Wound Healing Agent) in Terms of Acute Dermal Toxicity, Acute Dermal Irritation and Skin Sensitization

**DOI:** 10.4103/0971-6580.75847

**Published:** 2011

**Authors:** Amol R. Padol, K. Jayakumar, N. B. Shridhar, H. D. Narayana Swamy, M. Narayana Swamy, K. Mohan

**Affiliations:** Department of Pharmacology and Toxicology, Veterinary College, KVAFSU, Hebbal, Bangalore, India; 1 Department of Veterinary Pathology, Veterinary College, KVAFSU, Hebbal, Bangalore, India; 2 Department of Veterinary Physiology, Veterinary College, KVAFSU, Hebbal, Bangalore, India

**Keywords:** Draize test, erythema, irritation, sensitization

## Abstract

Acute dermal toxicity study was conducted in rats. The parameters studied were body weight, serum biochemistry and gross pathology. The animals were also observed for clinical signs and mortality after the application of test film. The dermal irritation potential of silk protein film was examined using Draize test. In the initial test, three test patches were applied sequentially for 3 min, 1 and 4 hours, respectively, and skin reaction was graded. The irritant or negative response was confirmed using two additional animals, each with one patch, for an exposure period of 4 hours. The responses were scored at 1, 24, 48 and 72 hours after the patch removal. Skin sensitization study was conducted according to Buehler test in guinea pigs, in which on day 0, 7 and 14, the animals were exposed to test material for 6 hours (Induction phase) and on day 28, the animals were exposed for a period of 24 hours (Challenge phase). The skin was observed and recorded at 24 and 48 hours after the patch removal. In acute dermal toxicity study, the rats dermally treated with silk film did not show any abnormal clinical signs and the body weight, biochemical parameters and gross pathological observations were not significantly different from the control group. In acute dermal irritation study, the treated rabbits showed no signs of erythema, edema and eschar, and the scoring was given as “0” for all time points of observations according to Draize scoring system. In skin sensitization study, there were no skin reactions 24 and 48 hours after the removal of challenge patch, which was scored “0” based on Magnusson/Kligman grading scale.

## INTRODUCTION

Silks are generally defined as protein polymers that are spun into fibers by *Lepidoptera* larvae such as silkworms, spiders, scorpions, mites and flies. Due to its special chemical structure and chemical composition, silkworm silk is highly compatible and absorbed easily with the human skin. Silk is a natural protein that is made up of 25–30% sericin and 70–75% fibroin proteins.[1] Silk is a natural protein polymer that has been approved as a biomaterial by the US Food and Drug Administration (FDA).[2] The silk sericin and fibroin proteins are prospective wound healing agents and are antioxidants and considered as bio-adhesive mediators of human body[3] Furthermore, silk fibroin is considered to be proper for the generation of biomedical products such as blended materials because of its minimal adverse effects on the immune system.[4] Silk protein is claimed to have wound healing property and efforts are being made to develop the wound dressing from silk proteins. The present study was designed to evaluate the acute dermal toxicity, dermal irritation and sensitization potential of the silk protein film. In the assessment and evaluation of the toxic characteristics of a substance, determination of acute dermal toxicity is useful where exposure by the dermal route is likely. It provides information on the health hazards likely to arise from a short-term exposure by the dermal route. This test also provides information on dermal absorption and the mode of toxic action of a substance by dermal route.

Dermal irritancy evaluation of topical agents was widely examined using Draize test, described by John Draize. Skin sensitization (allergic contact dermatitis) is an immunologically mediated cutaneous reaction to a substance. The sensitivity and ability of tests to detect potential human skin sensitizers are considered important in a classification system for toxicity relevant to public health.

## MATERIALS AND METHODS

The test material for the present study was obtained from a commercial firm (M/s. Sericare, Division of Healthline Pvt. Ltd., Bangalore, India). The test material was composed basically of silk proteins.

### Acute dermal toxicity study

This test was conducted as per the OECD Guideline 402.[5] Healthy adult Wistar albino rats, females weighing around 150–160 g and males weighing between 200 and 250 g, were used in the study. The females used were nulliparous and non-pregnant. Animals were acclimatized to the experimental conditions for 1 week. They were maintained under standard laboratory hygienic conditions, providing laboratory animal feed and water *ad libitum*. Animals were divided into four groups (*n* = 6) as follows: group I (control group male rats), group II (silk protein film applied male rats), group III (control group female rats) and group IV (silk protein film applied female rats).

Approximately 24 hours before the test, the hair coat was removed by closely clipping the dorsal area of trunk of the animals. Care was taken to avoid abrading the skin and only animals with intact skin were used. Films under study were moistened with physiological saline and applied on the shaved area. They were held in contact with the skin using porous gauze dressing and non-irritating tape for a period of 24 hours, whereas for the control groups, gauze moistened with physiological saline was applied and held in contact in a similar way as the treatment groups. Animals were observed within 30 min, 4 and 24 hours after the removal of films and kept under observation for 14 days. Individual weight of animals was determined on the day of application of film and weekly thereafter. They were observed for the changes in skin, eyes and mucous membranes, behavioral patterns, diarrhea, salivation and tremors. Mortality was recorded during the course of study. At the end of the study, survived animals were weighed, sacrificed and subjected to gross necropsy. In rats, the blood samples were collected on day 0, 7, 14 by retro-orbital plexus puncture method and serum biochemical parameters were estimated from the serum samples using clinical chemistry analyzer.

### Acute dermal irritation study

This test was conducted as per the OECD Guideline 404 (2002)[6] with a preferred sequential testing strategy. Healthy, young, adult, 4–6 months old, New Zealand White male rabbits, weighing around 1.0–2.0 kg were used as test system. Animals were individually housed and acclimatized to the experimental conditions for 1 week. Animals were free to access food and water *ad libitum*. Approximately 24 hours before the test, hair coat was removed by closely clipping the dorsal area of the trunk of the animals. Care was taken to avoid abrasion of the skin and only animals with intact skin were used for the study.

### Initial test (*in vivo* dermal irritation test using one animal)

Three test patches were applied sequentially, after observing that there was no serious skin reaction produced by the previous patch, at three different sites. The first patch was removed after 3 min and was observed for skin reactions, if any. Similarly, the second patch and third patch was removed after 1 and 4 hours, respectively, and observed for skin reactions. Later, the animals were observed for 14 days after the removal of patches, unless corrosion developed at an earlier time point indicating the need for immediate termination of the test.

### Confirmatory test (*in vivo* dermal irritation test with additional animals)

If there was no serious skin reaction observed in the initial test, the irritant or negative response was confirmed using up to two additional animals, each with one patch, for an exposure period of 4 hours. The animals were observed for 14 days after the removal of patches.

All the animals were examined for signs of erythema, eschar and edema. The responses were scored at 1, 24, 48 and 72 hours after the patch removal.

### Skin sensitization study

The present study was carried out to evaluate the skin sensitization potential of silk protein film as per EPA (Environmental Protection Agency) Health Effects Test Guidelines, OPPTs 870, 2600.

Healthy, young, adult male guinea pigs, weighing around 300–450 g, were divided into two groups (*n* = 6). The grouped animals were individually housed and acclimatized to the experimental conditions for 1 week. They were maintained under standard laboratory hygienic conditions, providing laboratory animal feed and water *ad libitum*. Approximately 24 hours before the test, hair coat was removed by closely clipping the dorsal area of the trunk of the animals.

#### Induction phase

On day 0, the animals (groups I and II) were clipped on the left flank to an area of 2 × 2 cm. The test film was moistened with physiological saline and held in contact with the skin of the animals in treated group using an occlusive patch for 6 hours. Similarly, a sterile gauze piece moistened with physiological saline was topically applied as control to the group I animals. On day 7 and 14, the experimental animals were exposed to the same application for 6 hours.

#### Challenge phase

On day 28, occlusive patch was retained for 24 hours. The experimental animals were challenged with the test film on untreated side of the flank. On day 29, the patch was removed and the whole flank was clipped.

All the skin samples (in terms of erythema and swelling) and systemic reactions resulting from induction and challenge procedures were observed and recorded at 24 and 48 hours after the patch removal according to the Magnusson/Kligman grading scale.

### Statistical analysis

Mean values and standard error of mean were calculated and expressed as Mean±SE. The data were analyzed by two-way analysis of variance (ANOVA) followed by Bonferroni’s post-test.

## RESULTS

In the acute dermal toxicity, there were no appreciable clinical signs observed throughout the observation period of 14 days after the patch removal and there were no appreciable changes in skin, eyes, mucous membranes and behavioral pattern. There was no mortality seen throughout the observation period. Also, there was no significant (*P*>0.05) difference in body weight in the treatment group compared to the respective control group female rats [Table 1]. Serum obtained from blood samples collected on day 0, 7 and 14 of the study period was used to estimate aspartate transaminase (AST), alanine transaminase (ALT), creatinine and blood urea nitrogen (BUN) for all the groups [Tables 2–5] respectively. None of the biochemical parameters differed significantly (*P*>0.05) in the treatment group from the control group in both male and female rats. Also, there were no prominent gross lesions observed in vital organs like liver, lung, heart, kidney and spleen of the treatment and control groups in both the sexes.

**Table 1:** Effect of treatment on body weight (g) in male and female rats during acute dermal toxicity study

	Males						Females					
	Control			Treatment			Control			Treatment		
	Day 0	Day 7	Day 14	Day 0	Day 7	Day 14	Day 0	Day 7	Day 14	Day 0	Day 7	Day 14
A_1_	210	216	219	215	220	222	178	180	192	182	184	189
A_2_	223	229	231	210	214	217	186	190	193	190	194	199
A_3_	217	220	223	227	231	233	180	183	187	179	184	188
A_4_	213	217	220	223	225	228	188	191	194	183	188	191
A_5_	224	227	231	219	222	225	190	194	197	186	188	192
A_6_`	208	213	217	222	227	230	179	184	189	191	194	200
Mean±SE	215.84±2.73	220.34±2.61	223.50±2.50	219.34±2.49	223.17±2.42	225.84±2.36	183.50±2.09	187.00±2.23	192.00±1.78	185.17±1.93	188.67±1.84	193.17±2.09

Values are expressed as Mean±SE; *n* = 6, *P*>0.05

**Table 2:** Effect of treatment on serum aspartate transaminase concentration (U/l) in male and female rats during acute dermal toxicity study

	Males						Females					
	Control			Treatment			Control			Treatment		
	Day 0	Day 7	Day 14	Day 0	Day 7	Day 14	Day 0	Day 7	Day 14	Day 0	Day 7	Day 14
A_1_	155.50	159.32	156.78	159.43	149.58	155.56	154.60	161.20	151.23	145.50	151.93	158.00
A_2_	157.60	160.85	152.65	159.76	154.45	159.04	161.14	154.54	164.41	154.70	161.32	154.51
A_3_	154.35	155.45	160.11	154.70	160.70	154.96	154.87	156.65	151.89	158.59	158.61	153.95
A_4_	158.89	154.40	149.43	161.42	157.97	156.76	144.59	151.61	158.59	149.59	160.74	160.69
A_5_	155.93	152.21	155.76	158.96	159.09	160.54	149.89	160.00	152.43	153.98	157.91	159.00
A_6_	158.87	159.49	154.00	153.47	158.66	161.60	152.49	150.64	158.68	145.50	151.93	158.00
Mean±SE	156.86±0.77	156.96±1.40	154.79±1.50	157.96±1.29	156.75±1.67	158.08±1.12	152.93±2.26	155.74±1.77	156.20±2.14	152.48±2.26	158.11±1.67	157.24±1.31

Values are expressed as Mean±SE; *n* = 6, *P*>0.05

**Table 3:** Effect of treatment on serum alanine transaminase concentration (U/l) in male and female rats during acute dermal toxicity study

	Males						Females					
	Control			Treatment			Control			Treatment		
	Day 0	Day 7	Day 14	Day 0	Day 7	Day 14	Day 0	Day 7	Day 14	Day 0	Day 7	Day 14
A_1_	45.59	47.11	44.18	45.60	44.72	44.83	48.95	44.57	49.69	44.52	50.96	45.70
A_2_	44.72	43.34	45.80	48.86	47.68	47.84	47.58	49.60	50.22	53.44	46.09	49.33
A_3_	47.94	48.51	48.62	49.85	49.54	44.12	43.50	43.57	51.02	43.42	48.01	48.50
A_4_	45.21	45.62	45.84	43.45	43.45	45.78	48.93	52.44	43.47	47.90	44.54	43.62
A_5_	50.64	46.51	49.65	44.96	44.81	43.42	49.63	50.68	45.70	45.12	49.96	49.11
A_6_	48.76	45.89	47.98	45.69	47.58	48.62	51.64	44.21	44.11	45.97	44.62	46.65
Mean±SE	47.15±0.96	46.17±0.71	47.02±0.85	46.41±0.99	46.30±0.95	45.77±0.85	48.38±1.12	47.52±1.57	47.37±1.36	46.73±1.48	47.37±1.12	47.16±0.92

Values are expressed as Mean±SE; *n* = 6, *P*>0.05

**Table 4:** Effect of treatment on serum creatinine concentration (mg/dl) in rats during acute dermal toxicity study

	Males						Females					
	Control			Treatment			Control			Treatment		
	Day 0	Day 7	Day 14	Day 0	Day 7	Day 14	Day 0	Day 7	Day 14	Day 0	Day 7	Day 14
A_1_	0.52	0.55	0.60	0.59	0.54	0.51	0.56	0.62	0.54	0.59	0.55	0.54
A_2_	0.63	0.60	0.56	0.58	0.59	0.53	0.59	0.54	0.60	0.64	0.65	0.64
A_3_	0.60	0.59	0.57	0.55	0.61	0.55	0.62	0.55	0.57	0.53	0.58	0.60
A_4_	0.55	0.53	0.62	0.59	0.54	0.51	0.54	0.53	0.54	0.59	0.53	0.50
A_5_	0.57	0.51	0.51	0.58	0.59	0.53	0.63	0.65	0.56	0.52	0.64	0.53
A_6_	0.56	0.59	0.59	0.55	0.61	0.55	0.58	0.62	0.59	0.55	0.52	0.59
Mean±SE	0.58±0.02	0.57±0.01	0.58±0.02	0.55±0.01	0.58±0.01	0.56±0.02	0.59±0.02	0.59±0.01	0.57±0.02	0.57±0.02	0.58±0.03	0.57±0.03

Values are expressed as Mean±SE; *n* = 6, *P*>0.05

**Table 5:** Effect of treatment on blood urea nitrogen concentration (mg/dl) in male and female rats during acute dermal toxicity study

	Males						Females					
	Control			Treatment			Control			Treatment		
	Day 0	Day 7	Day 14	Day 0	Day 7	Day 14	Day 0	Day 7	Day 14	Day 0	Day 7	Day 14
A_1_	39.90	41.62	40.50	43.42	43.80	41.34	39.55	38.43	40.64	45.17	43.04	44.07
A_2_	43.72	38.40	37.45	38.52	44.53	44.57	44.32	42.21	41.18	43.49	41.22	43.40
A_3_	45.59	44.40	45.09	47.21	39.67	41.25	42.13	39.49	40.08	44.73	39.69	41.15
A_4_	37.54	47.39	42.47	41.37	37.82	42.34	41.17	40.15	42.19	42.49	43.59	45.15
A_5_	44.12	42.23	38.04	49.59	38.02	38.16	44.37	41.09	43.55	48.69	47.17	49.10
A_6_	43.49	45.69	40.08	39.45	45.38	39.59	45.21	47.15	42.49	45.17	43.90	45.59
Mean±SE	42.40±1.24	43.29±1.32	40.60±1.17	43.26±1.80	41.54±1.40	41.21±0.90	42.80±0.89	41.42±1.27	41.69±0.53	44.96±0.87	43.10±1.05	44.75±1.09

Values are expressed as Mean±SE; *n* = 6, *P*>0.05

In the dermal irritation study, dermal reactions were graded according to Draize[7] and recorded at 1, 24, 48 and 72 hours after the patch removal. In the initial test, there was no skin reaction observed in rabbits after any of the three sequential (3 min, 1 and 4 hour) exposures. After a period of 14 days of observation, there was no appreciable skin reaction seen. In the confirmatory test, there was neither erythema nor edema seen in the treated animals and reaction was graded as “0” at all time points of observations.

In the skin sensitization study, skin reactions were read and graded 24 and 48 hours after the patch removal according to the Magnusson/Kligman grading scale. The dermal reactions were recorded and graded individually for each animal. No skin reactions were observed on the skin of guinea pigs in the treatment group after the challenge period, i.e., at 24 and 48 hours of patch removal, which was scored 0 based on the Magnusson/Kligman grading scale. Also, there were no systemic reactions observed after the induction and challenge exposure.

## DISCUSSION

In the assessment and evaluation of the dermal safety of a substance, acute dermal toxicity and the determination of irritant and sensitization effects on skin are important initial tests.

Serum biochemical parameters, viz., AST, ALT, BUN and creatinine did not show any statistically significant alterations in silk protein based film applied on male and female rats when compared to the respective control group animals, indicating that the test compound did not have any effect on serum biochemical parameters estimated. At autopsy, none of the treated and control group rats showed any gross pathological lesions. This lends support to the above findings, and thus, it could be concluded that silk protein based film did not exert any damage to vital organs when applied dermally. Acute dermal toxicity with sericin cream revealed no difference between the body weights of rats in sericin cream and cream base treated groups and there was neither mortality nor any gross lesions observed after sacrificing the animals, and thus, it could be concluded that sericin did not cause an appreciable dermal toxicity in rats.[8]

In the dermal irritation study, there were no signs of erythema, eschar and edema observed in the film treated group. The scoring was given as “0” for all time points of observation in the treatment group animals according to the Draize scoring system. These findings are in accordance with the reports of Pavankumar,[9] wherein the scoring was 0 at all time points of observation based on Draize scoring system when silk protein based films were applied on New Zealand white rabbits. Similarly, Thai silk soap does not cause irritation to the skin of laboratory rabbits when tested at a 10% concentration.[10] The silk protein is a biodegradable and highly biocompatible material. Thus, it has not caused any undue effect after application.[11] This suggests that silk protein film was not having dermal irritation potential in New Zealand white rabbits.

The guinea pigs in treatment group in skin sensitization study did not show any changes on the surface of the skin at 24 and 48 hours of patch removal and the reaction was graded as “0”. Moreover, silk fibers used as sutures (FDA approved) are biocompatible and less immunogenic and inflammatory than collagens or polyesters such as polylactic acid.[12] The results of present investigation correlate with the study conducted by Pavankumar,[9] wherein slight erythema was observed on the skin of the guinea pigs in silk protein based film treated group including control, which was scored “0” based on the scoring system given by Buehler.[13] This suggests that silk protein film is not having appreciable skin sensitizing property in guinea pigs.

It could be concluded from the present study that the test materials used in the study are safe under acute dermal toxicity, acute dermal irritation and skin sensitization.
